# THE CANONICAL WNT PATHWAY IN GASTRIC CARCINOMA

**DOI:** 10.1590/0102-672020180001e1414

**Published:** 2019-01-07

**Authors:** Levindo Alves de OLIVEIRA, Celina Tizuko Fujiyama OSHIMA, Pedro Augusto SOFFNER, Marcelo de Souza SILVA, Rodrigo Rego LINS, Andréa Cristina de Moraes MALINVERNI, Jaques WAISBERG

**Affiliations:** 1 Interdisciplinar Program in Surgical Sciences; 2 Laboratory of Molecular and Experimental Pathology, Department of Pathology, Federal University of São Paulo, UNIFESP/EPM, São Paulo, SP;; 3 Department of Surgery, ABC Medical School, Santo André, SP, Brazil

**Keywords:** Gastric carcinoma, Wnt signaling pathways, Immunohistochemistry, Signal transduction., Neoplasias Gástricas, Vias de Sinalização Wnt, Imunoistoquímica, Transdução de Sinal

## Abstract

**Background:**

It is believed that the Wnt pathway is one of the most important signaling involved in gastric carcinogenesis.

**Aim:**

To analyze the protein expression of canonical and non-canonical Wnt pathways in gastric carcinoma.

**Method:**

The immunohistochemistry was performed in 72 specimens of gastric carcinomas for evaluating the expression of Wnt-5a, FZD5, GSK3β, axin, CK1, ubiquitin, cyclin D1 and c-myc.

**Results:**

There were significant differences for cytoplasm and nucleus ubiquitin for moderately and well differentiated tumors (p=0.03) and for those of the intestinal type of the Lauren classification (p=0.03). The absence of c-myc was related to Lauren’s intestinal tumors (p=0.03). Expression of CK1 in the cytoplasm was related to compromised margin (p=0.03). Expression of cyclin D1 protein was more intense in male patients (p=0.03) There was no relation of the positive or negative expression of the Wnt-5a, FZD5, GSK3 and Axin with any clinicopathological variables.

**Conclusion:**

The canonical WNT pathway is involved in gastric carcinoma.

## RESUMO

**Racional:**

Acredita-se que a via Wnt é uma das mais importantes da sinalização envolvidas na carcinogênese gástrica.

**Objetivos:**

Analisar a expressão das proteínas das vias Wnt canônicas e não-canônicas no carcinoma gástrico e relacionar sua expressão com as variáveisclinicopatológicas.

**Método:**

Foram coletadas 72 amostras de carcinoma gástrico, e áreas representativas do tumor foram selecionadas para o Tissue Microarray. Imunoistoquímica foi realizada para avaliar a expressão de Wnt-5a, FZD5, GSK3β, axina, CK1, ubiquitina, ciclina D1 e c-myc.

**Resultados:**

Houve diferenças significativas para a expressão de ubiquitina no citoplasma e núcleo para tumores moderadamente e bem diferenciados (p=0,03) e para aqueles do tipo intestinal da classificação de Lauren (p=0,03). A expressão negativa da proteína c-myc no citoplasma foi relacionada aos tumores intestinais de Lauren (p=0,028). A expressão positiva de CK1 no citoplasma das células neoplásicas foi relacionada a tumores com margens cirúrgicas livre de envolvimento neoplásico (p=0,03). A expressão positiva da proteína ciclina D1 foi maior nos tumores dos homens (p=0,03). Não houve relação da expressão positiva ou negativa das proteínas Wnt-5a e FZD5 no citoplasma ou núcleo com quaisquer variáveis clinicopatológicas. O mesmo foi observado para GSK3β e Axin.

**Conclusões:**

A relação da expressão das proteínas da via canônica com as variáveis epidemiológicas e tumorais sugere sua participação na carcinogênese gástrica. Por outro lado, a ausência da relação das expressões das proteínas da via não-canônica sugere sua não participação na carcinogênese gástrica.

## INTRODUCTION

Gastric carcinoma has high global incidence and low average survival in both developed and developing countries. Despite several advances in conventional therapy, recurrence rates remain high and survival rates low^1^ ^,^ ^7^ ^,^ ^11^ ^,^ ^12^ ^,^ ^16^ ^,^ ^17^ ^,^ ^26^ ^,^ ^30^ ^,^ ^35^.

Changes such as mutations, deletions, inactivation by viruses and bacteria, and hypermethylation were involved in the onset of gastric cancer^7^ ^,^ ^16^ ^,^ ^28^ ^,^ ^32^. Wnt genes encode signaling proteins and are found in the genomes of all animals. Signaling is initiated when ligands of Wnt pathway are attached in a complex consisting of a receptor of frizzled family and a member of the family of low density lipid receptors^24^. The key molecule of the cascade is the cytoplasmic betacatenin protein whose stability is regulated by the so-called “destruction complex”^7^ ^,^ ^16^ ^,^ ^32^.

Wnt proteins play an important role in embryogenesis and maturation of tissues. These proteins act as ligands to components of frizzled family that are transmembrane cellular receptors. Once attached to these receptors, Wnt proteins can activate two distinct pathways of cell signaling: canonical and non-canonical pathways. Several proteins are involved in both of them^14^ ^,^ ^17^ ^,^ ^32^. 

Often, the Wnt signaling pathway is involved in gastric carcinogenesis and several proteins of this pathway may be mutated or expressed atypically in gastric tumor tissue. However, the involvement and mechanisms of Wnt pathway in the onset of gastric cancer are not fully understood as in colorectal cancer^8^ ^,^ ^32^.

A previous study from our research group suggested that the WNT/β-catenin pathway may be involved in gastric cancer. Lins et al. (2016)^24^ analyzed the expression of E-cadherin, beta-catenin, APC, TCF-4 and survivin in gastric cancer tissues by immunohistochemistry and verified relationship between the expression of the proteins and age of the patients and the anatomopathological aspects of gastric carcinoma as location, Lauren classification and degree of tumor penetration into the gastric wall. 

In order to identify another Wnt pathway proteins in these same samples we propose the study of two non-canonical pathway proteins (Wnt-5a, FZD5) and six canonical pathways proteins (GSK3B, axin, CK1, ubiquitin, cyclin D1 and MYC) and relate their expression with epidemiological and anatomopathological characteristics of the tumor.

## METHODS

The Ethic Review Committee at Federal University of São Paulo (UNIFESP), São Paulo, SP, Brazil, approved the study protocol (Registration no 1.128.919/2015).

A total of 72 specimens of primary gastric carcinomas (GC) were collected from patients who underwent radical surgical resection at the Department of General Surgery of ABC Medical School, SP, Brazil, from January 2007 to December 2010. The patients’ medical records were reviewed to determine their age, gender, anatomical site, tumor size, histological grade and the presence or absence of lymphatic, vascular or neural invasion. The inclusion criteria were patients aged over 18 years, of both gender, whom had undergone curative or palliative gastrectomy without neoadjuvant radio or chemotherapy, with histological examination confirming gastric adenocarcinoma. 

### TMA construction 

TMA blocks, also called receptor block, were constructed at the Laboratory of Molecular and Experimental Pathology, Department of Pathology, Federal University of São Paulo using the paraffin blocks containing the tissue of gastric cancer from the Department of Pathology, ABC Medical School. Representative areas selected by a pathologist of the 72 gastric carcinomas were selected from H&E stained sections. The selected area was marked in the respective paraffin block. A cylindrical core was created in the receptor block using Beecher™ equipment (Beecher Instruments, Silver Spring, MD, USA). A 1 mm cylinder of tissue was extracted from the selected area of the donating block and was transferred to the core in the receptor block. New core positions were created in the receptor block, separated by fractions of 1 mm such that a collection of tissue samples was created following the matrix arrangement.

### Immunohistochemistry

It was performed at Experimental Molecular Pathology Laboratory I Department of Pathology for evaluating the expression of Wnt-5a, FZD5, GSK3B, axin, CK1, ubiquitin, cyclin D1 and c-myc proteins according Gomes et al. (2011)^13^; da Silva et al. (2013)^10^.

Sections of 3 µm obtained from the TMA blocks were mounted on 3-aminopropylotrimetoxy-silane coated slides (Sigma), dewaxed in xylene, taken through ethanol to water to rehydrate. For antigen retrieval slides were placed in 0,01M citrate-buffer pH 6.0 and heated in a steamer for 30 min. Endogenous peroxidase activity was blocked by incubating the sections in a solution of 3% hydrogen peroxide for 20 min at room temperature. After these procedures, the sections were incubated with Wnt-5a (AF645) (1:100) and FZD5 (AF1617) purchased from R & D Systems, Inc, Minneapolis, MN, USA), GSK3β (sc-9166) (1:200); axin (sc-14029) (1:100); CK1 (sc-74582) (1:100); cyclin D1 (sc-8396) (1:100); c-myc (sc-40) (1:1000) and ubiquitin (sc-8017) (1: 150) purchased from Santa Cruz Biotechnology, Dallas, TX, USA) at 4º C overnight. The sections were washed with PBS and allowed to react with LSAB+ System-HRP (Biotinylated Link Universal) (Streptavidin-HRP) (Dako North America Inc.) for 30 min. Finally, staining was carried out using Liquid DAB+substrate chromogen system (Dako North America, Inc.) lightly counterstained with Harris hematoxylin and cover slipped with Entellan (Sigma). Negative and positive controls were made to run simultaneously. Positive control was represented by colon adenocarcinoma tissue. Negative controls were made by eliminating the primary antibody.

### Interpretation of reaction results

The staining patterns (membrane, cytoplasm and nucleus) were analyzed according to the criteria of distribution and intensity of staining. This analysis was semi-quantitative. A numerical scoring system with two categories was used to assess protein expression. The intensity of the staining was classified as negative (0 point), weak (1 point), moderate (2 points) and strong (3 points). The extent of the positive immunostaining area was classified as less than 10% (0 point), 11-25% (1 point), 26-50% (2 points) and above 50% (3 points). The intensity of the reaction was multiplied by the extension of the staining and the results were categorized into a score of 0 to 9. The reactions with score ≥4 were considered as positive and those with a score <4 were considered negative^8^ ^,^ ^32^. To evaluate the expression of proteins was used the Eclipse 80i-Nikon microscope. Representative areas of gastric adenocarcinoma tissue were captured using a Sony camera under 400X. 

### Statistical analysis

Descriptive analysis of the qualitative variables was done by the distribution of absolute frequency (n) and relative frequency (%). The comparison between the expression of each protein was performed by the Fisher’s exact test and the value of p<0.05 was considered significant.

## RESULTS

Clinicopathological data from the patients with gastric cancer are summarized in Table 1. Forty-five cases were men and 25 cases were women with a mean age of 65 years. Forty-six tumors were from the proximal region while 39 tumors were larger than 5 cm. No compromised surgical margin was observed in 63 cases. Venous invasion was identified in 23 patients and lymphatic invasion in 39. Perineural invasion was present in 43 patients. In 38 the tumors were moderately or well differentiated and Lauren classification included 49 intestinal adenocarcinomas and 21 diffuse adenocarcinomas. TNM staging showed that more than 50% of the patients were in the advanced stage of the disease.

**TABLE 1:** Clinicopathological variables of samples

Variables		n (%)
Age	> 50	64 (90.3)
	≤ 50	7 (9.7)
Gender	Male	45 (64.3)
	Female	25 (35.7)
Tumor location	Distal	26 (36.1)
	Proximal	46 (63.9)
Tumor size	> 5 cm	39 (54.9)
	≤ 5 cm	32 (45.1)
Margin compromised	absent	63 (90)
	present	7 (10)
Venous invasion	absent	46 (66.7)
	present	23 (33.3)
Lymphatic invasion	absent	30 (43.5)
	present	39 (56.5)
Perineural invasion	absent	26 (37.7)
	present	43 (62.3)
Differentiation grade	well/moderate	38 (55.9)
	little/undifferentiated	30 (44.1)
T stage	0	1 (1.4)
	1	2 (4.2)
	2	17 (23.6)
	3	41 (56.9)
	4	10 (13.9)
N stage	0	24 (34.7)
	1/2/3	46 (65.3)
Lauren classification	Intestinal	49 (69.4)
	diffuse	21 (30.6)
N		
	0	24 (34,7)
	1/2/3	46 (65,3)
Lauren classification		
	Intestinal	49 (69,4)
	diffuse	21 (30,6)

n=sample number

**FIGURE 1:**
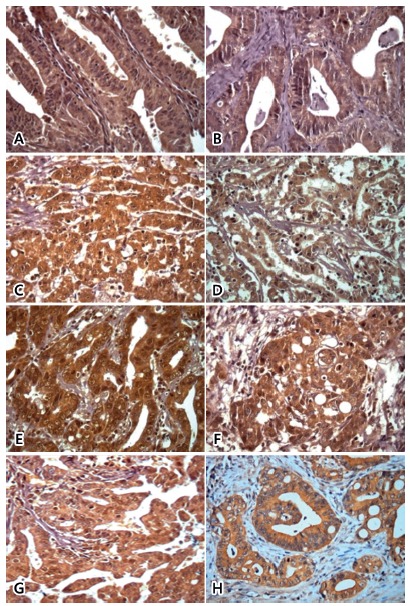
Photomicrography of immunohistochemistry results of proteins in gastric adenocarcinoma tissues: A=Wnt-5a; B=FZD5; C=GSK3β; D=axin; E=ubiquitin; F=ciclin D1; G=c-myc; H=CK1

Figure 1 shows photomicrography of immunohistochemistry results of Wnt-5a, FZD5, GSK3β, Axin, Ubiquitin, Cyclin D1, c-myc and CK1 proteins in gastric adenocarcinoma tissues.

n=sample number

Table 2 shows the presence or absence of Wnt-5a, FZD5, GSK3β, axin, CK1, ubiquitin, cyclin D1 and c-myc proteins in gastric adenocarcinoma tissues. No protein studied showed membrane marking.

**TABLE 2:** Presence or absence of Wnt-5a, FZD5, GSK3β, axin, CK1, ubiquitin, cyclin D1 and c-myc proteins in gastric adenocarcinoma tissues

Protein	Location	n	Positive (%)	Negative (%)
Wnt-5a	cytoplasm	71	24 (33.8)	47 (66.2)
	nucleus	72	59 (81.9)	13 (18.1)
FZD5	cytoplasm	72	2 (2.8)	70 (97.2)
GSK3β	cytoplasm	65	64 (98.5)	1 (1.5)
	nucleus	65	65 (100)	-
Axin	cytoplasm	67	63 (94)	4 (6)
	nucleus	67	67 (100)	-
CK1	cytoplasm	67	58 (86.6)	9 (13.4)
Ubiquitin	cytoplasm	67	62 (92.5)	5 (7.5)
	nucleus	67	62 (92.5)	5 (7.5)
Cyclin D1	nucleus	64	35 (64.7)	29 (45. 3)
c-myc	cytoplasm	66	16 (24.2)	50 (75.8)
	nucleus	70	30 (42.9)	40 (57.1)

There were significant differences for ubiquitin expression in the cytoplasm and nucleus for moderately and well differentiated tumors (p=0.03) and for those of the intestinal type of the Lauren classification (p=0.03). The negative expression of c-myc protein in the cytoplasm was related to Lauren’s intestinal tumors (p=0.028, Table 3). GSK3β, axin proteins expressions and nuclear expression of c-myc were not related to any clinicopathological variables. However, we note that axin protein expression in nucleus and cytoplasm was more intense in moderately well differentiated tumors and those of Lauren’s intestinal type but without statistical significance. Positive expression of CK1 in the cytoplasm of neoplastic cells was related to tumors showing a surgical margin free of neoplastic involvement (p=0.03). The positive expression of cyclin D1 protein was more intense in the tumors of male patients (p=0.03) but was not related to no other clinicopathological variables. There was no relation of the positive or negative expression of the Wnt-5a and FZD5 proteins in the cytoplasm or nucleus with any clinicopathological variables.

**TABLE 3:** Significant results of ubiquitin (cytoplasm/nucleus) and c-myc (cytoplasm) proteins in gastric adenocarcinoma tissues.

	Negative n (%)	Positive n (%)		p
Ubiquitin (cytoplasm)				
Differentiation grade	well/moderate	-	35 (100)	0,03*
	little/undifferentiated	4 (13.8)	25 (86.02)	
Lauren classification	Intestinal	1 (2.2)	45 (97.8)	0.03*
	Diffuse	4 (19)	14 (81)	
Ubiquitin (nucleus)				
Differentiation grade	well/moderate	-	30 (100)	0.04*
	little/undifferentiated	4 (13.8)	25 (86.02)	
Lauren classification	Intestinal	1 (2.2)	45 (97.8)	0.03*
	Diffuse	4(19)	14 (81)	
CK1 (cytoplasm)				
Margin committed	absent	6(10,2)	53(89,8)	0,03*
	present	3(50,0)	3(50,0)	
Ciclina D1 (nuclear)				
Gender	masculino	15(35,7)	27(64,3)	0,03*
	feminino	14(63,6)	8(36,4)	
c-myc (cytoplasm)				
Lauren classification	Intestinal	31 (67.4)	15 (32.6)	0.028*
	Diffuse	19 (95)	1 (5)	

n=number of samples; Fisher exact test*=significant

## DISCUSSION

The Wnt pathway is frequently involved in gastric carcinogenesis and the canonical pathway is considered the most important in its carcinogenesis. However, the non-canonical pathway has also been related to the genesis of gastric neoplasm^5^ ^,^ ^8^ ^,^ ^18^ ^,^ ^32^. 

The Wnt-5a protein is considered to inhibit the canonical signaling pathway and is considered a tumor suppressor. In the present study, the positive expression of Wnt-5a and FZD5 was not related to the variables studied. The immunohistochemical study of Kurayoshi et al.(2006)^22^ showed abnormal expression of Wnt-5a in 71 of 237 cases of gastric cancer. The positivity of Wnt-5a was correlated with advanced stages of the neoplasia and with the worse prognosis of the patients. Ara et al. (2016)^2^ evaluated the relationship of Wnt-5a to laminin g2, a protein related to cell adhesion and neoplastic invasion. The results suggested that Wnt-5a protein is involved in the progression of gastric cancer, a finding similar to that of other authors^15^ ^,^ ^29^ ^,^ ^33^. Zhang et al. (2015)^34^ showed that positive expression of Wnt-5a is related to the better prognosis of gastric carcinoma. On the other hand, FZD5 receptors receive canonical and non-canonical pathways signal, being important in the Wnt pathway. However, the FZD5 protein was more related to the non-canonical Wnt pathway^31^.

GSK3 is a multifunctional protein involved in mammalian cell regulatory pathways, including Wnt pathway, and it is part of the β-catenin destruction complex. In the present study, we identified a strong expression of the GSK3B protein in the nucleus and in the cytoplasm, but there was no relation to the epidemiological variables of the patients and anatomopathological of the tumor, which was also observed by other authors^31^ ^,^ ^34^. Cho et al. (2010)^9^ analyzed the expression of GSK3B in 281 cases of gastric carcinoma. Positive expression of this protein was related to early tumors, without lymph node metastases and without angiolymphatic invasion. 

The axin protein is part of the β-catenin destruction complex and acts as a tumor suppressor. Mutations in genes that synthesize axin are related to the genesis of hepatocellular carcinoma, endometrial adenocarcinoma and medulloblastoma^20^ ^,^ ^25^. In this study, axin protein expression was more intense in the nucleus than in the cytoplasm and was identified in 100% of the nuclei of gastric carcinoma cells. Cytoplasmic expression was also intense and predominated in moderately or well differentiated tumors and in intestinal type of Lauren’s classification^23^. Kim et al.(2014)^20^ showed that nine of 45 gastric carcinomas with microsatellite instability (MSI) had an AXIN2 frameshift mutation. Pan et al. (2008)^27^ analyzed 70 gastric carcinomas and identified 7.1% mutation in AXIN gene and concluded that these mutations contributed to gastric carcinogenesis. Mazzoni and Fearon (2014)^4^ reviewed the importance of axin and its variants in gastrointestinal carcinomas and observed that axin protein showed strongly positive expression in neoplastic cells. 

CK1 protein can be identified in the cell membrane, nucleus or cytoplasm. Seven members are known in the CK1 family in humans. CK1 epsilon has been related to the phosphorylation of Wnt pathway. There are few studies evaluating the expression of CK1 and its relation with the onset of gastric cancer. As part of the β-catenin destruction complex, positive protein expression suggests that it may be present in the onset stages of gastric carcinoma^4^ ^,^ ^21^. In the present study, a strong positive expression of CK1 was observed in the cytoplasm of the neoplastic cells where it normally exerts its function and not in the nucleus.

Ubiquitin is responsible for labeling protein to be degraded from the polyubiquitin chain. Regulating the plasma concentration of β-catenin as a member of Wnt pathway is performed by destruction complex together with ubiquitin. In the current study, the ubiquitin protein showed strong and identical expressions in the nucleus and cytoplasm. In both nucleus and cytoplasm, protein expression was significantly more intense in moderately or well differentiated tumors and in intestinal type of Lauren’s classification^23^.

Arici et al.(2009)^3^ evaluated the expression of cyclin D1 protein in the gastric mucosa and in the neoplastic gastric tissue. Expression of this protein was detected in 72% of gastric carcinoma cells and 55.8% of gastric mucosal cells. Such a finding suggests that cyclin D1 expression arises from the initial gastric lesions and tends to remain with tumor progression. We identified positive expression of cyclin D1 only in the nucleus of the neoplastic cells and there was no cytoplasmic expression of this protein. Greater number of tumors of the male patients presented positive expression than tumors of the female patients. Only nuclear expression was expected in view which cyclin D1 is a protein synthesized from stimulation of β-catenin in the nucleus. This finding suggests that canonical Wnt pathway is activated.

In the present study, c-myc protein expression was positive in cytoplasm in 24.2% and in nucleus in 42.9% of the cases. Negative cytoplasmic expression was significantly more frequent in intestinal type of Lauren’s classification^23^. Studies have shown that the amplification of the MYC gene in gastric cancer ranges from 38.1% to 40%. Khaleghian et al. (2016)^19^ evaluated the relationship of expression and amplification of the MYC gene with biodemographic and anatomopathological characteristics of the gastric carcinoma. In situ hybridization was positive in 43% and positive expression was identified in 14.7% of the cases. These authors identified the increased expression of MYC in diffuse tumors, unlike Calgano et al. (2013)^6^ who identified higher expression of MYC protein in intestinal type of Lauren’s classification. In addition, no difference was found in MYC protein expression in relation to tumor stages. Liu et al. (2014)^25^ verified the presence of positive MYC protein expression in 66.3% of gastric tumors. 

There are few studies involving these proteins using the immunohistochemical method and the results obtained are still unclear. However, expressions of the canonical Wnt pathway proteins were high in our samples showing the importance of these proteins. This result taken together creates perspectives for new studies in order to clarify the involvement of Wnt pathway in the onset of gastric carcinoma.

## CONCLUSION

The relation between the expression of canonical pathway proteins and epidemiological and tumor variables suggests their participation in gastric carcinogenesis. On the other hand, the absence of the expression of non-canonical pathway protein expression suggests its non-participation in gastric carcinogenesis.
